# A Prospective Nonrandomized Comparison of Wet Needling Versus Prolotherapy in Myofascial Pain

**DOI:** 10.7759/cureus.71427

**Published:** 2024-10-14

**Authors:** Neehara K Jacob, Ravi Sankaran

**Affiliations:** 1 Physical Medicine and Rehabilitation, Amrita School of Medicine, Kochi, IND; 2 Physical Medicine and Rehabilitation, Aster Medcity, Kochi, IND

**Keywords:** myofascial pain syndrome, myofascial trigger points, pain, prolotherapy, wet needling

## Abstract

Objective: The objective was to analyze the difference between prolotherapy and wet needling (WN) for myofascial trigger points (MTrPs) for the Visual Analog Scale (VAS), Oswestry Disability Index (ODI), Clinical Global Impression (CGI), and MTrP count.

Methods: Patients with myofascial pain for 1.5 years were included based on convenience sampling after a pilot study for sample size calculation. The WN group received an injection of bupivacaine 0.5% into the trigger points with WN. Participants in the dextrose prolotherapy (DPT) group received dextrose (25%) plus bupivacaine (0.5%) (1:1) into the same. Outcome measures were recorded at baseline immediate post-injection, one month, three months, and 24 months.

Results: Among the 200 participants, there was no significant difference in the baseline VAS score between the two groups nor for immediate post-VAS. At three months of follow-up, the mean VAS was 6.34 ± 1.44 in the WN group and 1.99 ± 0.89 in the DPT group (p = 0.03). The mean VAS score significantly changed in both groups but favored the DPT group (p = 0.001) and again at 24 months (p = 0.001). The ODI showed a similar trend favoring the DPT group at all intervals. On correlating the VAS score with the ODI score, a statistically significant correlation was seen at one month, favoring the WN group, and at the end of the third month, favoring the DPT group.

Conclusion: Both modalities are effective at one month. At three and 24 months, the DPT was significantly more effective in improving the VAS and ODI.

## Introduction

A recent analysis of the Global Burden of Disease data showed that approximately 1.71 billion people globally have musculoskeletal conditions [1]. Of the general population, 20%-50% may suffer this [2]. Myofascial pain syndrome (MPS) is a musculoskeletal disorder in which trigger points are found inside the muscle. It is a painful condition and is one of the common causes of healthcare visits [2,3]. Among the general population, the lifetime prevalence of MPS is 85% (with differences between genders) [4]. Its incidence is as high as 53% in women and 46% in men [5]. It is usually found between the ages of 25 and 50 [6,7]. Prior studies show that MPS is the most common cause of pain and was responsible for 54.6% of chronic head and neck pain and 85% of back pain [8].

Myofascial pain originates from taut, ribbon-like bands called the myofascial trigger points (MTrPs) in the muscle [9]. These are sensitized, palpable, areas of the muscle that are painful to manipulation, reproduce the patient's symptoms, and cause pain [10,11]. MPS is a chronic debilitating condition that requires long-term management and follow-up. The treatment includes inactivation of these and then restoring the normal muscle length, such as correction of the factors that caused or perpetuated the trigger points in the first place. Dextrose prolotherapy (DPT) and wet needling (WN) are the treatment options. They vary in that one induces macro-trauma (WN) to promote healing and the other creates controlled focal inflammation (DPT) to the same effect.

Objectives

Primary Objective

The primary objective was to analyze the Visual Analog Scale (VAS) in patients receiving WN or DPT.

Secondary Objective

The secondary objective was to analyze the Oswestry Disability Index (ODI) and MTrP count before and after the intervention in both groups and correlate the two variables per group.

## Materials and methods

This prospective nonrandomized study was conducted on patients with MPS. No changes were made after the initiation. It was conducted in the Department of Physical Medicine and Rehabilitation, from September 2020 to September 2022. It was registered (CTRI/2021/09/036393) and conducted per the WHO guidelines. Approval was obtained from the ethics committee, and the study was opened for enrollment. Convenience sampling was used for recruitment. Inclusion criteria were patient's age > 18 and <65, body mass index > 26 and < 35 kg/m^2^, with clinical presentation suggestive of MPS, a VAS score of >5 out of 10 per participant's self-assessment of pain on the first 30 minutes of walking in the morning, duration of at least 1.5 years, and presence of MTrPs and pain relapse despite prior treatment modalities, including physical therapy and nonsteroidal anti-inflammatory drugs (NSAIDs). Exclusion criteria were previous trauma, fibromyalgia, and other concomitant neurological and orthopedic conditions. Participants self-selected one of the two groups. Injections and follow-ups were performed by the first author, who was blinded. Injectate was mixed by a nurse not otherwise involved in the study.

Since no study could be located in the literature comparing the efficacy of a single injection of 25% dextrose plus 0.5% bupivacaine with a control group, a pilot study was conducted. Eleven cases and 10 controls were included in the pilot study. Based on the mean and SD of the difference in the VAS from baseline to one month between cases (6.82 ± 1.89) and controls (4.7 ± 2.06) observed in the pilot study conducted with 21 patients, and with 95% CI and 80% power, the minimum sample size was determined to be 42 per group.

At baseline, the VAS, ODI, Clinical Global Impression (CGI), and MTrP counts were noted. The muscles injected were the upper, middle, and lower trapezius and the erector spinae in the lumbar region. The muscles were injected with a 25-gauge 1-inch needle. The WN group received injections of bupivacaine (0.5%) into the trigger points (2 mL each) followed by 30 strokes of the needle after infiltration. The DPT group received prolotherapy using dextrose (25%) plus bupivacaine (0.5%) to a concentration of 18%. Both were taught home exercise programs based on the posture pattern as a maintenance program. At enrollment, the participants received two weeks of NSAIDs and neuropathic pain medicine. They were instructed to report any subsequent care they received for the study duration or any adverse effects (i.e., pain). Follow-ups were done at one, three, and 12 months as in-person visits. The total study duration was two years. 

Statistical analysis

The statistical analysis was done using IBM SPSS Statistics for Windows, Version 20.0 (Released 2011; IBM Corp., Armonk, New York, USA). For continuous variables, the results are given as mean ± SD, and for the categorical variables, they are given as frequency and percentage. To compare the mean difference of the numerical variables between the groups, an independent sample t-test was applied. To compare the pre- and post-findings of the numerical variables, a paired t-test was applied. To obtain the association of the categorical variables, a chi-squared test was applied. To test the statistically significant relationship between the continuous variables, the Pearson correlation coefficient was computed, and its statistical significance was tested using the linear regression t-test. A p-value of <0.05 was considered statistically significant.

## Results

**Figure 1:**
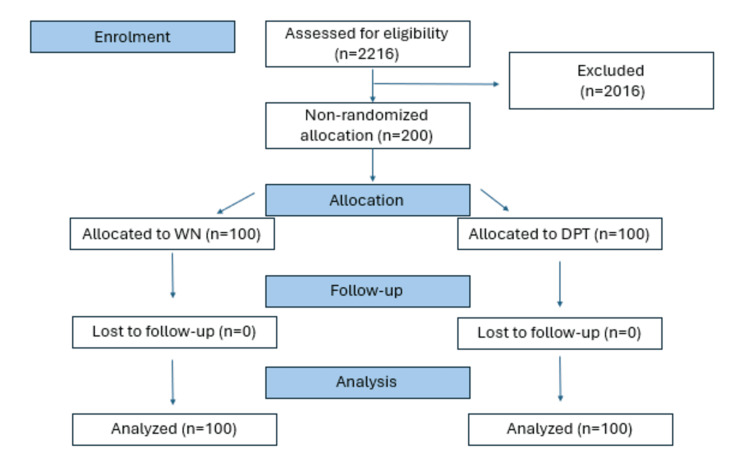
Participants' algorithm WN: wet needling, DPT: dextrose prolotherapy.

Among the 200 participants, 50% received prolotherapy and 50% received WN (see Figure 1). Of this, 56 were males (28%) and 144 were females (72%). Among the males, 24 (42.9%) were in the WN group and 32 (57.1%) were in the DPT group, whereas among the females, 76 (52.8%) were in the WN group and 68 (47.2%) were in the DPT group (see Figure 1). There was no significant difference in age and gender and no association of gender between the two groups (see Table 1). There were no reports of receiving subsequent care for the study period.

**Table 1:** Demographic details WN: wet needling, DPT: dextrose prolotherapy. The data have been represented as N, %, and mean ± SD. p-value is considered significant at <0.05.

	Groups		p-value (chi-squared)
	WN (n = 100)	DPT (n = 100)	
Male (56)	24 (42.9)	32 (57.1)	0.208
Female (144)	76 (52.8)	68 (47.2)	
Mean age	44.44 ± 14.07	46.47 ± 13.07	0.303 (0.04)

When comparing the groups at baseline, there were no significant differences between the two groups for the pre-VAS, immediate post-VAS, MTrP count, and baseline CGI. There was a significant difference for the baseline ODI (4.280, 95% CI 1.153-7.407, p 0.008) favoring the WN group. At one month, all measures improved significantly within each group. There was a statistically significant improvement of the ODI (5.400, 95% CI 3.086-7.714, p < 0.001) and CGI (0.210, 95% CI 0.118-0.302, p < 0.001) favoring the DPT group. At three months of follow-up, the VAS score (4.350, 95% CI 4.015-4.685, p < 0.001), the ODI score (25.840, 95% CI 23.548-28.132, p < 0.001), and the CGI score (3.780, 95% CI 3.675-3.885, p = 0.001) were again significantly different, favoring the DPT group. At 24 months, again there was a significant difference favoring the DPT group for the VAS, ODI, and MTrP counts (7 ± 6, 95% CI 1-13, p = 0.001) (see Table 2).

**Table 2:** Outcome variables WN: wet needling, DPT: dextrose prolotherapy, VAS: Visual Analog Scale, ODI: Oswestry Disability Index, CGI: Clinical Global Impression, MTrP count: myofascial trigger point count. The data have been represented as N, %, and mean ± SD. p-value is considered significant at <0.05.

	Groups		p-value (t-statistic)
	WN	DPT	
Pre-VAS	8.85 ± 1.23	8.89 ± 1.38	0.83 (-1.34)
Immediate post-VAS	7.59 ± 1.56	7.30 ± 1.57	0.19 (-2.21)
VAS one month	5.58 ± 1.26	5.19 ± 1.28	0.03 (-3.29)
VAS three months	6.34 ± 1.44	1.99 ± 0.89	0.001 (-28.1)
VAS 24 months	7.27 ± 2.11	2.56 ± 0.23	0.001 (-24.21)
Pre-ODI	46.9 ± 11.17	42.62 ± 11.26	0.008 (-3.67)
ODI one month	29.58 ± 8.54	24.18 ± 8.05	0.001 (-1.67)
ODI three months	35.6 ± 10.53	9.76 ± 4.83	0.001 (-23.65)
ODI 24 months	37.6 ± 6.31	12.21 ± 3.51	0.001 (-21.28)
Pre-CGI	4.94 ± 0.94	4.80 ± 1.03	0.30 (-1.56)
CGI one month	2.23 ± 0.42	2.02 ± 0.20	<0.001 (-4.65)
CGI three months	4.80 ± 0.49	1.02 ± 0.20	0.001 (-3.21)
MTrP baseline	45.46 ± 5.57	47.33 ± 7.61	0.74 (-12.91)
MTrP 24 months	21.68 ± 8.23	14.92 ± 11.51	0.001 (-7.82)

**Figure 2:**
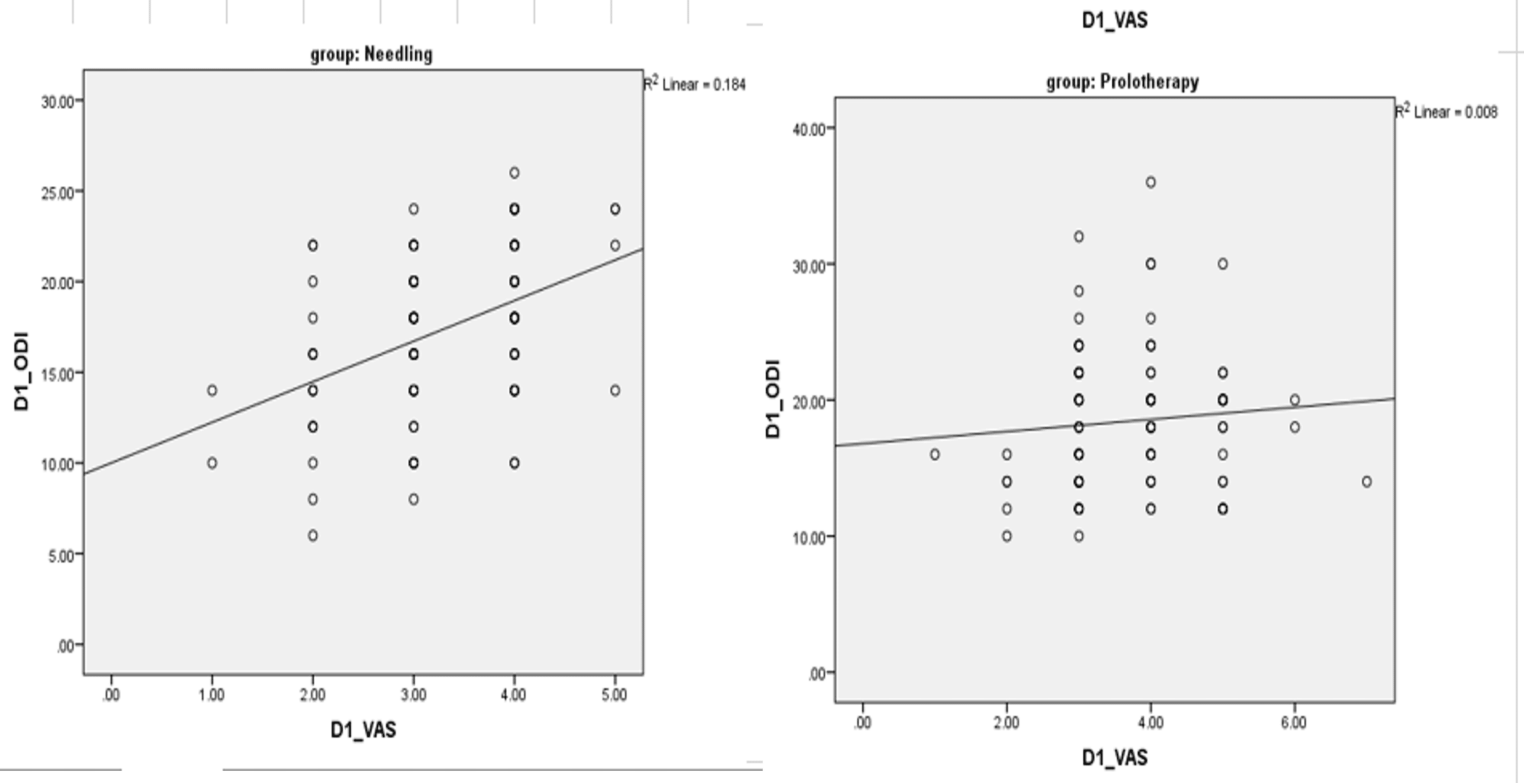
Correlation between variables DPT: dextrose prolotherapy, ODI: Oswestry Disability Index, VAS: Visual Analog Scale, WN: wet needling. Correlation plot showing a strong relationship between the VAS and the ODI changes for the DPT group, but not for the WN group.

On correlating the VAS score with the ODI score in the first month, it showed a moderate degree of positive correlation (r = 0.429) in the WN group and was found to be statistically significant with a p-value of <0.001 and a low degree of positive correlation (r = 0.087) in the DPT group and was found to be not statistically significant with a p-value of 0.389. On correlating the VAS score with the ODI score in the third month, it showed a low degree of positive correlation (r = 0.177) in the WN group and was found to be not statistically significant with a p-value of 0.078 and a moderate degree of positive correlation (r = 0.435) in the DPT group and was found to be statistically significant with a p-value of <0.001. Figure 2 shows a scatter diagram for the VAS with the ODI at the first month in the WN and DPT groups. The Pearson correlation was done to assess the correlation between the changes in the ODI and the VAS. The DPT group showed better ODI scores as the VAS reduced compared to the WN group (see Figure 2).

## Discussion

Both groups had a comparable number of trigger points and similar baseline VAS and CGI scores. The ODI was better at baseline for the WN group. There was no change in the pain scores immediately following intervention in both groups; however, there was a significant change in the scores at follow-up at both one and three months for both groups, favoring the DPT group. There was a significant drop in the pain scores for the DPT group in the third month compared to the WN group. The VAS score for the WN group at three months was greater than the scores of the first month, indicating a worsening. The CGI at three months again favored the DPT group, indicating affect, as rated by the clinician, was improving. When comparing the number of trigger points at 24 months, the DPT group had significantly less. On correlating the VAS score with the ODI score, a statistically significant correlation was seen at the end of the first month, favoring the WN group, and at the end of the third month favoring the DPT group. This reflects an improvement in the functional status of the participants as pain improves in the DPT group over the WN group. As pain cannot be objectively and reproducibly quantified, the CGI was used to cross-validate the patients' reports. The clinician appraised the scores matched with the reported outcomes. 

Classical prolotherapy is four sessions over eight weeks. This applies to ligamentous prolotherapy. We injected MTrP instead. The clinical value of a single dose was studied earlier [11]. This prolotherapy regimen group provides a single injection of hypertonic dextrose plus bupivacaine. Adding a long-acting local anesthetic agent provides longer pain relief. It is unlikely that the anesthetic contributed to the outcomes between groups, as both received it. The WN causes macro-trauma, which may not heal as well as the micro-trauma from the DPT. Hypertonic dextrose initiates an inflammatory cascade which includes proliferation then healing and subsequently tissue growth. This resulted in a clinical improvement due to the restoration of tissue integrity and is the reason why the VAS scores were overall better even up to 24 months.

The ODI score assesses the functional output of the enrolled participants. There was a reduction in the ODI in one month for both groups; however, the DPT group had a significant reduction compared to the WN group. The improved ODI, despite a worse VAS, is likely due to the reduction of the exacerbated pain at presentation. This became evident at the next interval. There was a significant reduction in the 3rd and 24th months for the ODI in the DPT group, which suggested an improved functional capacity. On the other hand, the increase in the ODI in the third month for the WN group suggested a decrease in functional capacity. This plateaued, which could be seen at 24 months. This is not an optimal outcome.

The CGI baseline scores of both groups were similar, and there was a reduction in the score at one month that was statistically significant, favoring the DPT group. There was a significant reduction of the CGI in the DPT group in the third month; however, for the WN group, the scores were comparable to the preinjection scores. Naturally, the clinician's rating of patient affect is higher in the group with less pain and better function.

The DPT has been used for chronic neck pain, shoulder pain, knee osteoarthritis, tendinopathies, and radiculopathies. There is a paucity of literature regarding the use of this for the treatment of MPS. In a randomized controlled trial (RCT) conducted for patients with chronic shoulder pain, classical/ligamentous prolotherapy was compared to the WN with lignocaine [12]. The follow-up in the ninth month showed that pain reduced more, favoring the DPT group. These results are comparable to our study, which showed a longer-term improvement.

A concern exists regarding the concentration of the solution to be used in the DPT. A RCT study compared 5% dextrose to normal saline to lignocaine. The VAS revealed that 5% dextrose improved the pain score on day seven, favoring DPT over the other groups [13]. This supports the “energy crisis” theory in which injections as low as 5% dextrose provide pain relief. Dextrose in MPS can be used for both short-term and long-term effects. The administration of the pericellular dextrose dose as small as 0.5% stimulates the production of fibroblastic cytokines. Ten percent dextrose can cause proliferation without inflammation. It increases the width of the connective tissue and the thickening of collagen [14].

A case series of patients with cervical, thoracic, and lumbar pain showed that 20% DPT injected into the affected facet joints had improved disability scores. Here, the patients received injections weekly for three weeks and for one month if needed. The patients who received these injections were willing to take multiple injections. These reduced their dependence on other modalities, increased their ability to work, and reduced the frequency of oral medications [15].

Is the agent of choice important when treating sacroiliac diseases? A study used levobupivacaine and triamcinolone versus dextrose with multiple injections [16]. There was an improvement of the pain scores by more than 50% in the DPT group over the others at two weeks. The reason for this could be the fact that they have provided biweekly injections.

MPS is not caused by a deficiency of the therapeutic modalities for pain. At some point, a person must stop the behaviors/repetitive strain patterns that lead to cumulative trauma. For this, a self-maintenance program is needed. This reduces the impact of repetitive strain patterns, which often resume once the pain is gone. Preventing patients from doing what made the pain occur in the first place is the final level of care and is not addressed in this protocol. As a bridge to that state, they were taught home exercises based on their posture pattern. Our limitations were as follows. The scales we utilized to measure the clinical improvement were subjective rather than objective. This was a single-center prospective study, and the assessors were aware of the procedures. The inclusion of multiple centers would have been an improvement along with double blinding. All study participants were administered a single injection of the drug at one single time point. Standard DPT requires multiple injections.

The limitations of our study include the absence of a placebo-controlled group, a small sample size, and a lack of comparison with other pharmacological agents. A RCT with a larger sample size, long-term follow-up, and comparison with pharmacological treatment is needed to gain more knowledge. As this study is a non-RCT, selection bias can occur. We tried to remove that by having all the options and details available.

## Conclusions

Both the DPT and the WN are effective in reducing pain in the short term. At 24 months, a single-dose prolotherapy was more effective than the WN. The changes were noted in the improved pain scores, disability index, and improved quality of life. 

The WN is, in general, a destructive process that introduces focal macro-trauma to induce healing. The DPT produces focal-controlled inflammation with agents designed to promote healing. This is why the DPT showed better results than the WN in the long-term follow-up.
